# Global Grassland Diazotrophic Communities Are Structured by Combined Abiotic, Biotic, and Spatial Distance Factors but Resilient to Fertilization

**DOI:** 10.3389/fmicb.2022.821030

**Published:** 2022-03-28

**Authors:** Maximilian Nepel, Roey Angel, Elizabeth T. Borer, Beat Frey, Andrew S. MacDougall, Rebecca L. McCulley, Anita C. Risch, Martin Schütz, Eric W. Seabloom, Dagmar Woebken

**Affiliations:** ^1^Department of Microbiology and Ecosystem Science, Centre for Microbiology and Environmental Systems Science, University of Vienna, Vienna, Austria; ^2^Department of Botany and Biodiversity Research, University of Vienna, Vienna, Austria; ^3^Department of Ecology, Evolution, and Behavior, University of Minnesota, St. Paul, MN, United States; ^4^Swiss Federal Institute for Forest, Snow and Landscape Research WSL, Birmensdorf, Switzerland; ^5^Department of Integrative Biology, University of Guelph, Guelph, ON, Canada; ^6^Department of Plant and Soil Sciences, University of Kentucky, Lexington, KY, United States

**Keywords:** grassland soil, *nifH* gene sequencing, seasonal climate, plant cover type, nutrient addition, nutrient network, nitrogen fixation, biogeography

## Abstract

Grassland ecosystems cover around 37% of the ice-free land surface on Earth and have critical socioeconomic importance globally. As in many terrestrial ecosystems, biological dinitrogen (N_2_) fixation represents an essential natural source of nitrogen (N). The ability to fix atmospheric N_2_ is limited to diazotrophs, a diverse guild of bacteria and archaea. To elucidate the abiotic (climatic, edaphic), biotic (vegetation), and spatial factors that govern diazotrophic community composition in global grassland soils, amplicon sequencing of the dinitrogenase reductase gene—*nifH*—was performed on samples from a replicated standardized nutrient [N, phosphorus (P)] addition experiment in 23 grassland sites spanning four continents. Sites harbored distinct and diverse diazotrophic communities, with most of reads assigned to diazotrophic taxa within the *Alphaproteobacteria* (e.g., *Rhizobiales*), *Cyanobacteria* (e.g., *Nostocales*), and *Deltaproteobacteria* (e.g., *Desulforomonadales*) groups. Likely because of the wide range of climatic and edaphic conditions and spatial distance among sampling sites, only a few of the taxa were present at all sites. The best model describing the variation among soil diazotrophic communities at the OTU level combined climate seasonality (temperature in the wettest quarter and precipitation in the warmest quarter) with edaphic (C:N ratio, soil texture) and vegetation factors (various perennial plant covers). Additionally, spatial variables (geographic distance) correlated with diazotrophic community variation, suggesting an interplay of environmental variables and spatial distance. The diazotrophic communities appeared to be resilient to elevated nutrient levels, as 2–4 years of chronic N and P additions had little effect on the community composition. However, it remains to be seen, whether changes in the community composition occur after exposure to long-term, chronic fertilization regimes.

## Introduction

Nitrogen (N) is a key nutrient in terrestrial ecosystems and is one of the limiting factors for primary production (Vitousek and Howarth, 1991; Vitousek et al., 2002; Fay et al., 2015). While part of the N-budget is continuously recycled through remineralization, new input of fixed N is essential to offset N losses and meet the N demands of terrestrial ecosystems. Biological dinitrogen (N_2_) fixation is a primary source of N input into ecosystems (Cleveland et al., 1999; Galloway et al., 2004) and can be performed by a group of bacteria and archaea—the diazotrophs. The genetic ability to fix N_2_ spans numerous taxonomic groups; in addition, this functional guild encompasses various microbial lifestyles (including autotrophs and heterotrophs; aerobes and anaerobes; free-living and symbiotic microorganisms; Zehr et al., 2003; Raymond et al., 2004; Boyd and Peters, 2013). The phylogeny of diazotrophs has been investigated using a highly conserved marker gene encoding for the dinitrogenase reductase protein, the *nifH* gene. It has been shown that diazotrophs can be grouped into four main phylogenetic clusters, partly reflecting which metal co-factors are embedded in the nitrogenase enzyme or the different life strategies used by the microorganism (Zehr et al., 2003; Raymond et al., 2004; Gaby and Buckley, 2011).

Grasslands are one of the largest ecosystems on Earth, covering around 37% of the ice-free land surface and providing the living environment for around 14% of humanity in 1995 (White et al., 2000; IPCC, 2019). As the productivity of grasslands depends on continuous N inputs, diazotrophic communities are vital to this ecosystem (Reed et al., 2011). Studies investigating potential drivers of diazotrophic communities have primarily focused on the local scale (e.g., single sites), testing few environmental variables (such as ambient carbon, N, phosphorous levels, or N fertilization), or broad climate variables [such as mean annual temperature (MAT) or mean annual precipitation (MAP); Wang et al., 2017a,b; Feng et al., 2018; Lin et al., 2018; Han et al., 2019]. However, despite increasing knowledge about factors structuring diazotrophic communities within sites, we are still lacking an understanding of environmental key factors that structure these communities on a global scale. This includes the potential for significant spatial turnover within diazotrophic communities, regionally or continentally, with unclear implications on their function.

Moreover, long-term climate variables (e.g., MAP and MAT), and the effect of seasonal climate conditions in combination with nutrients and the plant community on diazotrophic communities in the world’s grasslands remains understudied. Such multifactorial interactions have been shown to drive soil fungal diversity (Rillig et al., 2019). Understanding the environmental factors that govern the distribution of diazotrophs is crucial for grassland management, especially in light of the expected climate change-induced alteration of precipitation and temperature regimes (IPCC, 2019) and anthropogenic elevated nutrient levels (Bennett et al., 2001; Erisman et al., 2013), concomitant with changes in plant community composition, such as decreasing legume cover (Tognetti et al., 2021). Environmental changes are expected to also affect soil microbial communities through the proliferation of species with better adaptation to the novel conditions (e.g., De Vries and Shade, 2013). A global change factor with a high potential to alter the diazotrophic community composition is anthropogenic nutrient addition, particularly additions of N and phosphorous (P). These nutrients can be limiting for primary production globally (Fay et al., 2015) and are thus applied to terrestrial ecosystems on a large scale to improve productivity (Mackenzie et al., 1998; Galloway et al., 2004; Bouwman et al., 2009).

Our overarching hypothesis was that the diazotrophic communities in grassland soils globally are not shaped by a single factor but rather by a combination of abiotic, biotic, and spatial factors. Furthermore, we expected that climate seasonality would correlate better with diazotrophs than annual averages due to the potential for rapid population dynamics in bacteria. We predicted that the abundance of certain plant functional groups (grasses, legumes) shape the diazotrophic community composition, as specific associations between diazotrophs and *Poaceae* and *Fabaceae* are known (Reinhold-Hurek and Hurek, 1998; Carvalho et al., 2014). Spatially, we expected distance-based discontinuity of diazotrophic community composition (i.e., closer sites more similar than distant sites), which could reflect processes most constrained by geographic distance (e.g., dispersal; Telford et al., 2006), or those determined by abiotic conditions (e.g., environmental sorting; Van Der Gucht et al., 2007). In addition, the availability of nutrients (such as N and P) were predicted to govern the diazotrophic community composition, as N and P supply can alleviate potential nutrient limitations and are therefore known to influence the diazotrophic community composition in agricultural and plantation soils directly (Feng et al., 2018; Lin et al., 2018; Wang et al., 2018) or to impact N_2_ fixation activity (Dynarski and Houlton, 2018; Wang et al., 2018).

In our study, we explored abiotic, biotic, and spatial factors that potentially shape the diazotrophic community composition in grassland soils spanning a global range of edaphic, climatic, vegetative, and spatial variables using amplicon sequencing of the *nifH* gene. The similarity of the diazotrophic community composition among grasslands on four continents was compared to key nutrient and seasonal climate factors, various annual and perennial plant covers, and the geographic distance to determine the extent to which each or a combination of factors are associated with changes in this community.

## Materials and Methods

### Site Characteristics and Experimental Design

The sites used for this study were part of the Nutrient Network (NutNet)—a globally replicated experiment investigating the effects of nutrient addition and herbivores on grassland ecosystems (Borer et al., 2013). In this study, samples from a set of 23 sites were used. The sites span temperate-zone regions of Africa (three sites), Australia (one site), Europe (three sites), and North America (17 sites), thus capturing a globally relevant range of edaphic and climatic conditions. The locations and abbreviations of the site names are listed in Supplementary Table S1. The sites include a wide range of grassland types (e.g., annual grasslands, mesic grasslands, old fields, and montane meadows) which we broadly refer to as “grasslands” here. The sites vary along biotic and abiotic gradients of climate, edaphic properties, plant community composition, and spatial distance. For each site, precipitation and temperature data were extracted from Bioclim V1.4 (WorldClim; Hijmans et al., 2005). Soil pH, total soil nutrients (e.g., C, N, and P), and soil texture were measured as described in Prober et al. (2014). The plant community composition and abundance of different plant functional groups (e.g., annual and perennial grass, forb, and legume covers) were determined by site-level experts (Prober et al., 2014). Additionally, the aridity index representing the ratio between annual precipitation and estimated vegetation water demand for all sites was extracted from CGIAR Consortium for Spatial Information (Trabucco and Zomer, 2018). A standardized nutrient addition experiment was replicated at each site for 2–4 years before investigating the diazotrophic communities (Borer et al., 2013). Soils of three plot replicates from each of five different treatments were sampled during local plant growing season for this study: nitrogen addition (+N), phosphorus addition (+P), nitrogen and phosphorus addition (+N/+P), no-addition control, and no-addition fenced control plots to exclude vertebrate herbivores like ruminants. The two no-addition treatments were used to study the baseline native diazotrophic community structure across the 23 grassland sites. Both N and P were applied once per year before the plant growing season at a concentration of 10 g m^−2^ (100 kg ha^−2^) as timed-release urea [(NH_2_)_2_CO] and triple-super phosphate [Ca(H_2_PO4)_2_] (Borer et al., 2013).

### PCR Amplification and Sequencing

The composition of the diazotrophic community was quantified using the DNA extracts from Leff et al. (2015), which investigated bacterial, archaeal, and fungal communities. We amplified the marker gene for N_2_ fixation (*nifH*) for subsequent amplicon sequencing on an Illumina MiSeq platform (Illumina, San Diego, CA, United States). A two-step approach was used to amplify and barcode samples as described previously in Herbold et al. (2015), with few modifications. Depending on the measured DNA concentration of soil extracts using Quant-iT PicoGreen dsDNA Assay (Thermo Scientific, Waltham, MA, United States), 10 ng or at most 4 μl of DNA-template was used per first-step PCR reaction. To target the *nifH* gene, IGK3 (5′ GCI WTH TAY GGI AAR GGI GGI ATH GGI AA 3′) as forward and DVV (5′ ATI GCR AAI CCI CCR CAI ACI ACR TC 3′) as reverse primer were used (Ando et al., 2005) to amplify a 340 bp *nifH* gene fragment, which were shown to cover a larger diversity of *nifH* than other primer pairs, especially in phylogenetic clusters III and IV (Angel et al., 2018). The first-step PCRs were done in triplicate reactions of 50 μl, using the following program: 94°C for 5 min followed by 30 cycles of 94°C for 30 s, 52°C for 45 s, and 72°C for 45 s, and a single step of final elongation at 72°C for 10 min. In the second-step PCR, 5 μl of the pooled purified (using ZR-96 DNA Clean-up Kit; Zymo, Irvine, CA, United States) first-step PCR product was amplified using the headed-barcode primer and the same cycle program except for only using 15 cycles. In cases where the PCR reactions indicated saturation on agarose gel electrophoresis (very strong bands, no remaining primers), the reactions were repeated using 5 cycles less in the first-step PCR and also 5 cycles less in the second-step PCR. One negative control sample using water instead of template DNA was included in every performed PCR. In addition, PCR from extraction blanks (resulting from DNA extractions using water instead of soil) was performed to ensure no contamination during DNA extractions. PCR products were purified using AMPure XP magnetic beads (Beckman Coulter, Krefeld, Germany) in a bead/sample ratio of 0.7 to exclude primer dimers of around <100 bp. The total fragment size including primer, head, and barcode sequences was around 440 bp. Illumina Truseq library preparation and MiSeq sequencing were performed by Microsynth (Balgach, Switzerland) in the 2 × 300 cycle configuration using the MiSeq Reagent kit V3 (Illumina, San Diego, CA, United States). The raw reads were deposited in the NCBI Short Read Archive under the accession number PRJNA777635.

### Classification of *nifH* Genes and Community Analyses

Raw MiSeq amplicon reads were processed with the previously developed NifMAP pipeline (Angel et al., 2018). In short, the primer pair IGK3-DVV covers the largest *nifH* diversity (especially in Clusters III and IV) but is also prone to co-amplify homologous genes to the *nifH*. Therefore, Hidden Markov models (HMMs) were used to determine the similarity of every read or OTU to a gene-specific reference alignment and thus filtered out non-*nifH* data. Initially, a nucleotide-based HMM filtered the assembled contigs to keep *nifH*-like reads only. Following chimera check and OTU clustering (3% radius), specific HMMs were used to filter OTUs of homologous genes to *nifH* (*bchX*, *chlL*, *bchL*, and *parA*) at the amino acid level. Information on removed reads and OTUs during sequence data processing is documented in Supplementary Table S2. OTU representatives were taxonomically classified using BLASTP (Altschul et al., 1997) against the RefSeq database (Pruitt et al., 2007) and assigned to phylogenetic clusters of *nifH* using classification and regression trees (CART; Frank et al., 2016).

All downstream analyses were carried out using R (version 3.5.2; R Core Team, 2018) and were plotted using the package ggplot2 (version 3.3.3; Wickham, 2016). Unless otherwise mentioned, all functions came from the R package vegan (version 2.5-6; Oksanen et al., 2019). For data manipulation and function-wrapping, the phyloseq package was used (version 1.26.1; McMurdie and Holmes, 2013). Bray–Curtis dissimilarity measure was calculated for all community distance matrices. As a cutoff, any sample with less than 500 *nifH* reads (i.e., after removing homologs) was excluded from the analysis. As a result, of the originally 25 sites investigated in Leff et al. (2015), two study sites (Mt. Caroline, Australia; Shortgrass Steppe LTER, United States) were entirely removed from analyses in this study. The dataset comprised in total 6,826 OTUs and on average 2,200 reads per sample. To study the native diazotrophic community structure across grassland sites data of the no-addition treatment types were used and consisted of 5,257 OTUs assigned to 241 genera.

Variance partitioning analysis was done using permutational multivariate ANOVA (PERMANOVA; 10^4^ permutations; McArdle et al., 2001), which is implemented in the vegan function adonis(). The effect of read number per sample (library size) on the differences in community composition (Bray–Curtis dissimilarity) was significant but explained only 0.9% of the variance in the data. Consequently, no data transformation for library size normalization was used, except conversion to relative abundance for diazotrophic community analyses. PERMANOVA was also used for testing potential differences in communities between the open and fenced control plots and between nutrient addition regimes (under block design, with permutations within each site). Subsequently, per site pairwise plot comparisons were conducted using PERMANOVA tests of subsetted 23 datasets. Value of *p* were corrected according to Benjamini–Hochberg (BH) for multiple testing (Benjamini and Hochberg, 1995). To derive geographic distances between study sites from GPS coordinates, the function distm() of the R package geosphere (version 1.5-10; Hijmans, 2019) was used. The correlation between the geographic distribution of sites with the diazotrophic community composition was tested using Mantel test (geographical distance matrix in km; function mantel()) and PERMANOVA (categorical variables: site, region, continent), and visualized using constrained distance-based Redundancy Analysis (dbRDA) by the function capscale() including the continent and study site as factors. Core microbiome analyses were performed using the function core() of the package microbiome (version 1.5.28; Lahti and Shetty, 2017).

Mantel tests were performed to calculate correlations between the diazotrophic community dissimilarity matrix and a distance matrix for each environmental variable separately, with subsequent value for *p* correction after BH for multiple testing. We focused first on identifying the environmental variables that would correlate with beta diversity at the OTU level. Subsequently, we reduced the taxonomic resolution and clustered all reads based on the assigned genera (function tax_glom() in the phyloseq package) to decrease the high heterogeneity in the diazotrophic community compositions across samples. We used Spearman’s *ρ* as a measure of correlation between the OTU/genera dissimilarity matrix and the Euclidean distance matrix of each abiotic factor and biotic plant cover variable and the Bray–Curtis dissimilarity index for the plant communities (total plant community and functional subgroups). Abiotic environmental variables were normalized ( $\left(x-\bar{x}\right)/\sigma$ ) using the function scale() in the R Base package, except for vegetation cover since this was expressed as a percent of ground cover. The plant cover of the grass family *Poaceae* was separately analyzed from other graminoid families (*Cyperaceae* and *Juncaceae*)—from here on referred to as “grass-like”—as associations between diazotrophic bacteria and some members of *Poaceae* are known from the literature (Reinhold-Hurek and Hurek, 1998; Carvalho et al., 2014). The most informative variables were implemented in models using the MRM() function for multiple regression on matrices (Lichstein, 2007) from the R package ecodist (version 2.0.1; Goslee and Urban, 2007) and revised based on forward selection due to the statistical output. To assess if correlations between the diazotrophic community composition and spatial distance reflect solely similar environmental conditions in sites closer to each other, a linear model of the geographic (in km) and the environmental distance between samples was calculated (lm() function in the lme4 package, version 1.1-23; Bates et al., 2015). The environmental distance matrix between samples was formed by combining normalized (scale() function) previously identified most predictive abiotic and biotic variables using the Euclidean distance. Correlations between the relative abundance of certain genera or phylogenetic *nifH* clusters (merged OTUs to the four clusters by tax_glom() function) and environmental variables were tested by calculating linear models (lm() function). Constrained dbRDA ordinations for environmental and fertilization variables were performed with the function capscale(). Differential abundance analysis of the effect of nitrogen addition was performed using corncob (version 0.2.0; Martin et al., 2020). Only sites that showed significant changes due to N addition at the community level using PERMANOVA were tested (sites: look.us and konz.us). OTUs that appeared in <2.5% of the samples were discarded prior to analysis. The test was performed using the differentialTest() function while controlling for dispersion. Analysis scripts, as well as edaphic and plant metadata are available at https://github.com/mnepel/nutnet_grassland_diazotrophs.

## Results

### Geographic Distance Increased Dissimilarity in the Diazotrophic Community

**Figure 1:**
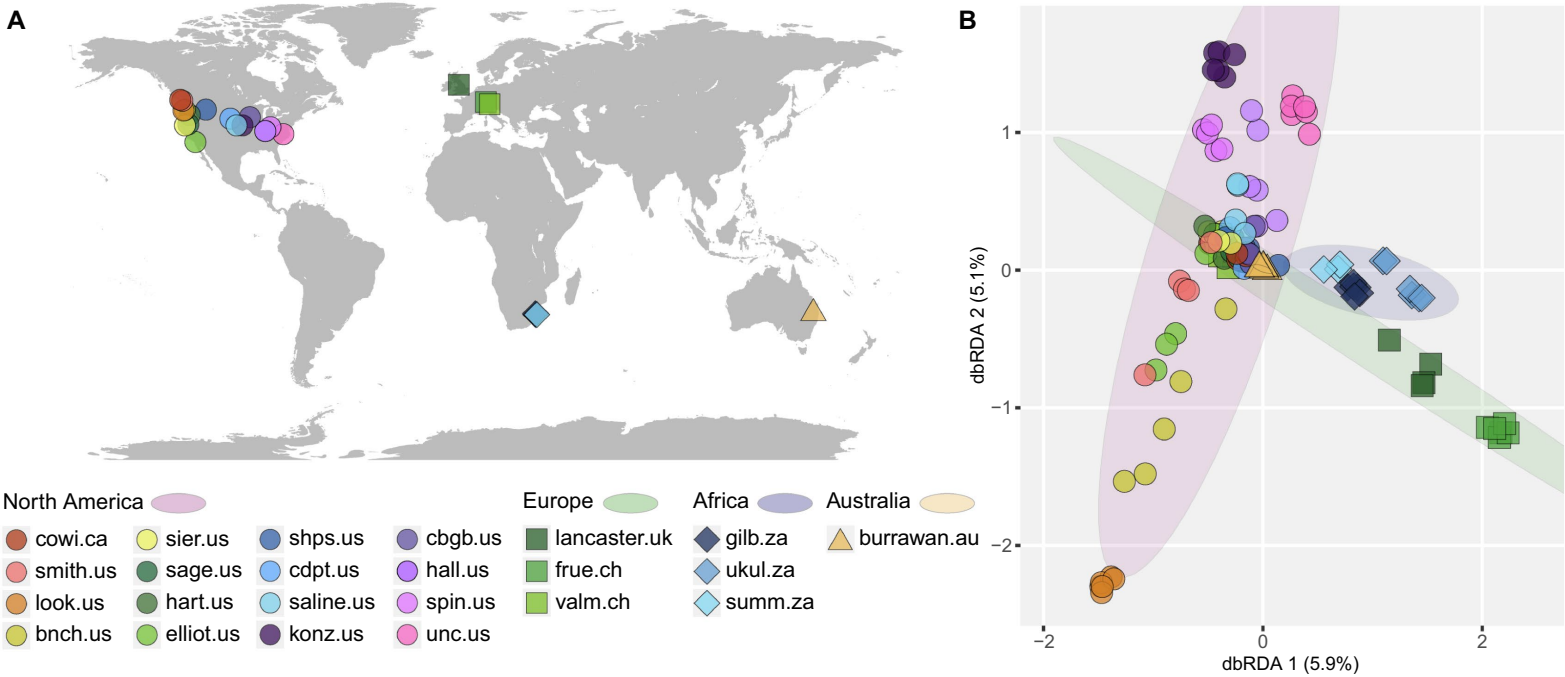
**(A)** Global distribution of all studied temperate grassland soils, **(B)** distance-based redundancy analysis (dbRDA) displaying the effect of study sites on the Bray–Curtis dissimilarity of diazotrophic communities. Each color represents one collection site. Only samples from non-addition plots were used here. Shapes denote the respective continent and ellipses encompass sites from the same continent. More information on sampling sites and their geographic locations can be found in Supplementary Table S1.

Two no-addition treatment types were used to study the baseline native diazotrophic community structure across the 23 grassland sites, in four continents (Figure 1A). These control plots—fenced and unfenced—were merged as there were no significant community composition differences associated with fencing (*R*^2^ = 0.007, *p* = 0.973). The continent, region, and site location significantly correlated with the diazotrophic community composition, explaining incrementally 11%, 10%, and 31% (cumulatively 52%) of the diazotrophic compositional variation (all *p* = 0.001). Beta diversity of these control plots showed distinct community structure among sites, suggesting some influence of geographic distance (Figure 1B). There was some similarity among grassland diazotrophic communities in North America (Figure 1B, pink ellipse), which overlapped with samples of other geographic regions [Figure 1B, Europe (green ellipse), and Australia (triangles)]. Similar to these findings, a significant correlation existed between geographic distance and the diazotrophic community (Spearman’s *ρ* = 0.386, *p* < 0.001), indicating not only samples within a site but also sites within regions have more similar communities compared to other regions. The strongest predictor for diazotrophic communities was the study site itself, explaining 52% of the variation and indicating strong heterogeneity among sites. This was further supported by detecting only few OTUs that were shared among the sites. Nearly 3,000 of the 5,257 detected OTUs (55%) were found in only one site, whereas 88 OTUs (1.6%) were widespread (detected in at least 12 out of 23 sites) accounting for around 43% of total reads. Furthermore, only 13 OTUs were present across 90% of sites accounting for around 18% of total reads, and these were classified as *Nostocales* (*Calothrix*), *Rhizobiales* (*Bradyrhizobium*, *Hyphomicrobium*), *Rhodospirillales* (*Komagataeibacter*, *Skermanella*), and *Nitrosomonadales* (*Methyloversatilis*). Two OTUs, assigned to *Methyloversatilis* (“OTU32”) and *Bradyrhizobium* (“OTU88”), were present at all sites.

When reducing the taxonomic resolution to the genus level, diazotrophic beta diversity remained highly correlated with the site, explaining 58% of community variation (*p* < 0.001; dbRDA plot in Supplementary Figure S1), though the geographic distance did not correlate with the distribution of genera (*ρ* = 0.013, *p* = 0.322). Thirty-one (31) out of 241 detected genera (12.8%) were present in only one site, and 21 were found across all sites. Only four ubiquitous genera accounted each for at least 0.1% of reads per site. *Clostridium* (*Clostridiales*), *Bradyrhizobium* (*Rhizobiales*), *Nitrospirillum* (*Rhodospirillales*), and *Geobacter* (*Desulfuromonadales*) together made up around 30% of total reads.

**Figure 2:**
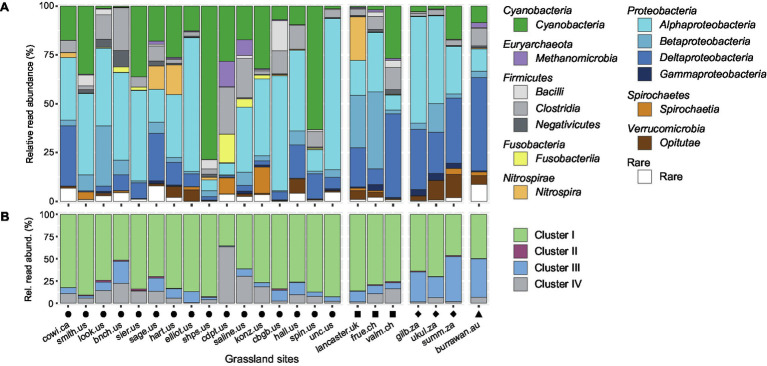
Diazotrophic community composition per grassland site. **(A)** Taxonomic affiliation of OTUs summarized at the class level based on BLASTP analysis; **(B)** relative read abundances of phylogenetic *nifH* clusters based on classification and regression trees (CART) analysis. On the *x*-axis, every bar represents the average community per study site, sorted according to longitudinal coordinates, whereas the *y*-axis shows the relative read abundance of taxonomic classes **(A)** or phylogenetic *nifH* clusters **(B)**. Shapes above the grassland site names denote the respective continent (circle: North America, square: Europe, diamond: Africa, triangle: Australia).

The distinct diazotrophic communities among sites, suggesting spatial or localized environmental sorting, were also apparent at the taxonomic class level. Based on the classification of *nifH* amino acid sequences, the relative read abundance of certain taxonomic groups varied considerably (Figure 2A). *Alphaproteobacteria* (average 34%, range 5.5%–77%), *Cyanobacteria* (19%, 0.5%–79%) and *Deltaproteobacteria* (15%, 0.5%–48%) were the most abundant bacterial classes, followed by *Clostridia* (8%, 1%–24%) and *Betaproteobacteria* (8%, 0.5%–39%). A higher proportion of *Cyanobacteria* reads were detected at the North American sites, whereas *Deltaproteobacteria* reads appeared to be more abundant at non-North American sites (Figure 2A). The three most abundant classes were mainly represented by reads assigned to *Bradyrhizobium* (*Rhizobiales*, *Alphaproteobacteria*; 16%, 0.5%–42%), *Calothrix* and *Nostoc* (*Nostocales*, *Cyanobacteria*; 10%, 0.1%–65%; 3.6%, 0.06%–20%, respectively) and *Geobacter* (*Desulfuromonadales*, *Deltaproteobacteria*; 8.4%, 0.3%–41%).

The *nifH* sequences were assigned to one of four phylogenetic clusters based on regression trees (Figure 2B), from which ecophysiological traits and the nitrogenase type can be inferred. Reads affiliated with cluster I were dominant among most sites (74%, 36%–93%; Figure 2B, in green), representing mainly the canonical Mo-Fe nitrogenase of aerobic *Proteobacteria* and *Cyanobacteria*. Cluster II reads, representing an alternative nitrogenase using only Fe as a co-factor, were hardly present (0.8%, 0%–2%; Figure 2B, in red). Even though the climatic and edaphic characteristics varied across grassland sites, none represented environmental conditions where the use of the alternative nitrogenase would be favored. Cluster III that consists of the canonical Mo-Fe nitrogenase mainly from anaerobic microorganisms (mainly *Deltaproteobacteria*, *Opitutae*, and *Clostridia*) were most prevalent in the African and Australian sites, along with one North American site (13%, 0.5%–51%; Figure 2B, in blue). In three sites (cowi.ca, sage.us, valm.ch), the high relative abundance of *Deltaproteobacteria* was not reflecting the relative abundance of cluster III nitrogenases, as almost all reads were assigned to *Geobacter* that possesses mainly a cluster I nitrogenase in our dataset. A significant positive correlation (*p* = 0.008) was seen between the read abundance of presumably anaerobic diazotrophs (cluster III and cluster I *Geobacter* reads) with the mean precipitation in the warmest quarter at study sites (Supplementary Figure S2). On average, 12% (0.7%–63%) of reads per site were affiliated with cluster IV, mainly found at some North American sites (Figure 2B, in gray), which was assumed to contain only inactive nitrogenases until recently (Zheng et al., 2015).

### Plant Communities, Climate, and Edaphic Factors Influence the Composition of Diazotrophs

Many environmental factors covaried with the composition of diazotrophs at the OTU level (Supplementary Table S3). Based on the probability and highest Spearman’s *ρ* values, the best abiotic model consisted of several climate and edaphic properties (Table 1). The mean temperature in the wettest quarter (Temp_WET_Q), mean precipitation in the warmest quarter (Precip_WARM_Q), soil C:N ratio, and soil texture (percent clay and sand) together explained 18% of the diazotrophic compositional variation (*p* < 0.001). Chemical soil properties like pH or ambient nutrient levels (e.g., C, N, P, K, Mn, or Fe) and long-term climate variables like MAP or the aridity index were less informative than seasonal climatic conditions. Since soil texture was only determined in half of all samples, a reduced model using only the two climate variables and soil C:N ratio (*R*^2^ = 0.172, *p* < 0.001) was used to integrate further vegetation properties.

**Table 1:** Most significant correlations (Spearman’s *ρ*) of the diazotrophic community of unamended treatment plots at the OTU level with abiotic environmental and biotic vegetation variables, using Mantel tests.

Variable	*ρ*	*p*
*Abiotic parameters*		
Temp_WET_Q	0.34	0.002
Precip_WARM_Q	0.25	0.002
Soil C:N ratio	0.25	0.002
Percent clay	0.29	0.002
Percent sand	0.24	0.002
*Biotic parameters*		
Perennial grass cover	0.23	0.005
Perennial grass-like cover	0.15	0.005
Perennial herb cover	0.12	0.005
Total plant community	0.08	0.012
Perennial grass community	0.27	<0.001
Perennial grass-like community	0.18	<0.001
Perennial forb community	0.25	<0.001

Value of *p* is corrected for multiple testing according to Benjamini–Hochberg.

**Figure 3:**
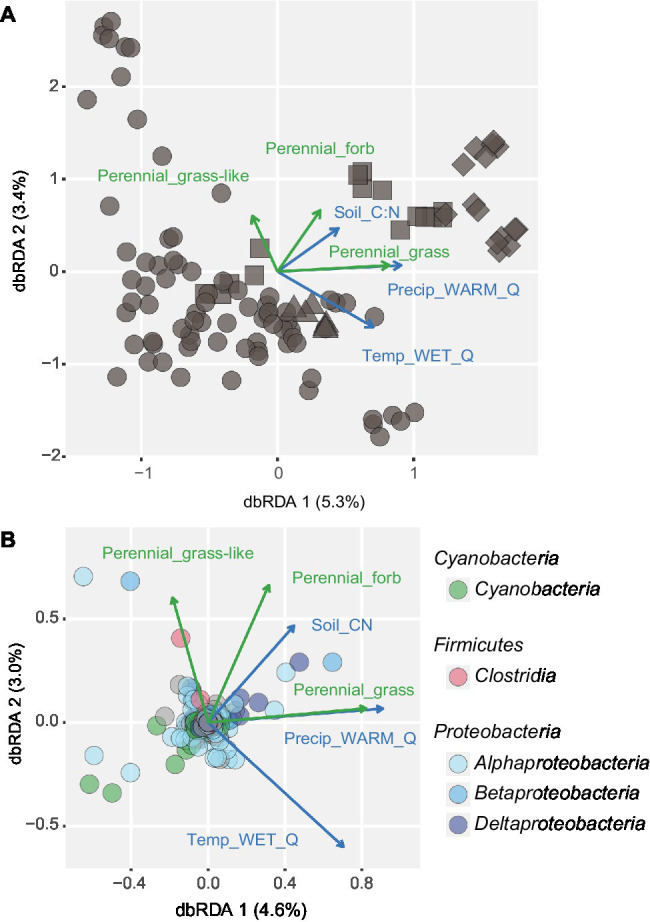
Distance-based redundancy analysis showing **(A)** the variation of the diazotrophic community and **(B)** the OTUs of relevant taxonomic classes as a factor of different environmental parameters based on our best descriptive model. Three abiotic variables, mean temperature in the wettest quarter (Temp_WET_Q), mean precipitation in the warmest quarter (Precip_WARM_Q), and the soil C:N ratio; and three plant cover variables, perennial grass, perennial grass-like, and perennial forb cover are depicted as blue or green vectors, respectively. Average environmental conditions per study site are listed in Supplementary Table S5. Shapes in panel **(A)** denote the respective continent (circle: North America, square: Europe, diamond: Africa, triangle: Australia). In panel **(B)**, colored circles denote OTUs assigned to the five most read-abundant taxonomic classes potentially shaped by displayed environmental factors. OTUs of other taxonomic classes are depicted in gray.

We tested how plant community composition and percent cover of different plant functional groups correlated with the diazotrophic community composition (Table 1; Supplementary Table S4). Perennial grass, perennial grass-like (*Cyperaceae* and *Juncaceae*), and perennial herb cover correlated significantly with the diazotrophic community, explaining 8% of the variation at the OTU level (*p* < 0.001). Diazotrophic beta diversity also correlated with the composition of total plants, perennial grasses, perennial grass-like, and perennial forbs (Table 1). In contrast, no correlations were detected between the diazotrophic community composition and legume, cryptic, or woody plant cover and their communities (Supplementary Table S4). The combination of best abiotic and biotic variables Temp_WET_Q, Precip_WARM_Q, soil C:N ratio, perennial grass cover, perennial grass-like cover, and perennial herb cover explained 19% of the diazotrophic compositional variation (*p* < 0.001; Figure 3A). The study site averages of these variables are listed in Supplementary Table S5. Among others, some OTUs assigned to *Cyanobacteria* (*Nostocales*) tended to be negatively correlated with forb and grass covers, whereas some *Deltaproteobacteria*-OTUs (*Syntrophobacterales*) were positively correlated with plant cover and a higher soil C:N ratio (Figure 3B). One OTU assigned to *Clostridia* (*Clostridiales*) positively correlated with grass-like plant cover, whereas the distribution of *Alphaproteobacteria* (mainly *Rhizobiales* and *Rhodospirillales*) displayed different OTUs correlating with different variables.

Similar to the OTU level analysis, many factors correlated with the diazotrophic composition at the genus level, but with lower Spearman’s *ρ* correlation values (Supplementary Tables S6, S7). The best predictive model consisted of edaphic, climatic, and plant variables, with soil pH, calcium concentration (Ca), copper concentration (Cu), mean precipitation in the coldest quarter (Precip_COLD_Q), and annual grass cover, explaining 14% of the variation (*p* < 0.001). The study site averages of these variables are listed in Supplementary Table S8. The corresponding constrained ordinations indicated correlations between environmental factors (as vectors) and certain diazotrophic genera (Supplementary Figure S3). Linear models verified significant correlations for six genera after subsequent value of *p* corrections for testing the effect of multiple factors per genus (Supplementary Table S9). With increasing soil pH and Ca, the genus *Calothrix* increased, and the genus *Bradyrhizobium* decreased in relative read abundance. *Geobacter* and *Skermanella* increased with Cu, whereas *Bradyrhizobium* decreased. The genus *Skermanella* also increased in relative read abundance with the amount of annual grass cover. One unclassified *Burkholderiales* taxus and *Azorhizobium* varied with site-level seasonality (Precip_COLD_Q).

### Effect of Elevated N and P on the Diazotrophic Community

**Figure 4:**
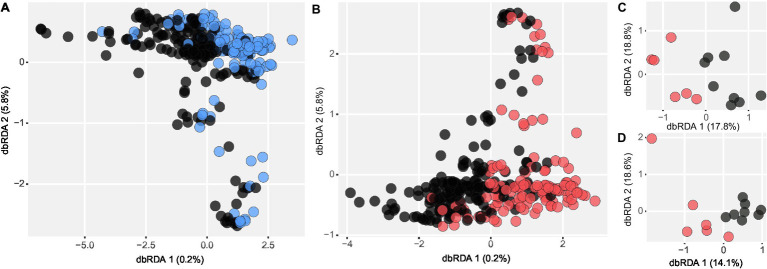
Distance-based redundancy analysis showing the variation of the diazotrophic community **(A)** due to P and **(B)** due to N addition. Significant community shifts detected in **(C)** konz.us and **(D)** look.us after 4 years of chronic N fertilization. Nutrient-treated samples are depicted in blue (P) and red (N); respective control samples are represented in black.

Experimental addition of nutrients for 2–4 years had little impact on the diazotrophic communities. The constrained ordination plot showed only a slight separation of P-treated samples from controls (Figure 4A), and PERMANOVA (including site as a random effect) confirmed the lack of significant differences (*R*^2^ = 0.002, *p* = 0.417). Similarly, the N treatment did not cause a consistent community shift across sites (*R*^2^ = 0.002, *p* = 0.178; Figure 4B). Notably, the N treatment did impact the diazotrophic communities but had differing effects at different sites (N treatment interacting with collection sites; *R*^2^ = 0.047, *p* = 0.035), in contrast to the P treatments that consistently induced no detectable change (*R*^2^ = 0.044, *p* = 0.534). Subsequent data subsetting and testing for each site revealed a slight but significant community shift in two North American sites, which experienced 4 years of chronic N addition, konz.us (*R*^2^ = 0.177, *p* = 0.046) and look.us (*R*^2^ = 0.141, *p* = 0.046). Constrained ordinations of both study sites showed a separation of samples according to N treatment (Figures 4C,D), displaying a community shift in N fertilized plots. Differential abundance analysis showed a significant change in the read abundance of certain OTUs (Supplementary Figure S4). Three OTUs assigned to *Cyanobacteria* and *Methanomicrobia* decreased, whereas one *Clostridia* OTU increased in relative read abundance. Some OTUs within *Proteobacteria* significantly increased and others decreased in relative abundance from control to N treated plots.

## Discussion

### Highly Diverse but Distinct Site-Specific Diazotrophic Communities

The soil diazotrophic community varied significantly across study sites, even though all are classified as temperate-zone grasslands. There was a very small shared diazotrophic microbiome detectable among the sites. The strong site effect in diazotrophic community composition is consistent with previous findings from these grasslands of general bacterial, archaeal, and fungal communities (Leff et al., 2015). We attribute this site effect to the wide range of climatic and edaphic conditions found among our sites, but likely in combination with some spatial distance factors (e.g., restricted dispersal). The heterogeneity in environmental conditions, including soils with, for example, varying nutrients and oxygen concentrations, as well as differing seasonality and vegetation, selects for diverse diazotrophic communities to allow for N_2_ fixation across a multitude of different environmental conditions. Additionally, the significant correlations of various spatial levels (e.g., geographic distance, region, and continent) with the diazotrophic community composition imply more similar community compositions at sites closer to each other than at sites that were further apart. This denotes a role of spatial factors or can mirror environmental conditions, which may be more similar in sites which are closer to each other. Comparing the geographic distance and the environmental distance (based on the six previously identified biotic and abiotic variables), we confirm that samples further apart experience more distinct environmental conditions (Supplementary Figure S5). However, due to the visible variation in environmental conditions, and as both distances are not fully collinear, one can see indications for not only environmental, but also spatial influence, on the community composition. Although an interplay of environmental variables and spatial distance is possible as shown in microbial communities of other environments (Wang et al., 2008; Schauer et al., 2010; Martiny et al., 2011), we cannot rule out the possibility that we lack environmental variables, which could explain the visible geographic pattern, or that more in-depth sequencing of *nifH* would diminish the heterogeneity among sites and thus the observed geographical pattern.

### Climatic and Edaphic Variables, Especially Water-Related, Govern the Diazotrophic Community

MAT and MAP were shown previously to influence diazotrophs in grassland soils (Wang et al., 2017a; Che et al., 2018). We also found significant relationships between these parameters and the diazotrophic communities across our grasslands; however, other climatic factors were stronger predictors, namely, mean temperature in the wettest quarter and mean precipitation in the warmest quarter. Water and temperature represent important factors for microbial life (Davey, 1989) and appear to shape the diazotrophic community. While the interplay between temperature and water availability is likely to determine microbial growth conditions in these grassland soils, the factor precipitation in the hottest season could additionally refer to the risk of drought stress and desiccation (Schimel, 2018) for diazotrophs. Our results also indicate that soil texture shapes the diazotrophic community, potentially indirectly by influencing the water content in soil. Higher clay content, if not dominating, results in increased water retention due to smaller pore sizes and, therefore, higher soil moisture over more extended periods, while a higher percentage of sand leads to rapid drainage of water (Kramer, 1983).

There are different possibilities as to why the soil C:N ratio correlated with the diazotrophic community composition. This ratio might reflect variations in the plant community, and a correlation was observed between diazotrophs and plants. Plants influence the soil C:N ratio *via* root exudation or indirectly through the production of plant polymeric C. Vegetation shaping soil bacterial communities *via* the C:N ratio was among others shown for tundra landscapes due to litter decomposition (Chu et al., 2011; Shen et al., 2013). Alternatively, the soil C:N ratio could reflect the state of organic C. A high soil C:N ratio might indicate a high abundance of organic C, which is known to further positively influence water retention in soils (Rawls et al., 2003). Interestingly, the effect of soil pH, which was previously shown to shape the diazotrophic community composition (e.g., Wang et al., 2017a; Che et al., 2018; Han et al., 2019), did not correlate with the OTU-based diazotrophic community composition in our study. One reason for that could be the high compositional heterogeneity due to the global scale in combination with the small variation in pH across sites. On genus level, when the heterogeneity is reduced, edaphic properties like soil pH and cations (Ca and Cu) were associated with the distribution of diazotrophic genera. In detail, read abundance of the genera *Calothrix* and *Bradyrhizobium* correlated with soil pH, concordant with earlier studies isolating *Calothrix* strains from alkaline (Pandey et al., 2005; Nayak and Prasanna, 2007; Rinkel and Manoylov, 2014) and *Bradyrhizobium* strains from acidic environments (Graham et al., 1994; Ozawa et al., 1999). Although Cu is an important co-factor for enzymes, it can have a toxic effect on bacteria (Trevors and Cotter, 1990; Ladomersky and Petris, 2015), which might explain the decrease of *Bradyrhizobium* read abundance. In contrast, *Geobacter* and *Skermanella* reads were positively correlated with Cu in our study. Previously, a *Geobacter* species was shown to survive on Cu (Kimber et al., 2020) and, a *Skermanella* strain resistant to the toxic metalloid antimony was isolated from a coal mine (Luo et al., 2012), suggesting that Cu tolerance is plausible in these groups. Precipitation in the coldest quarter, a water-related factor, determined the relative abundance of *Azorhizobium* and an unclassified *Burkholderia* group.

In conclusion, the most significant abiotic environmental factors that correlated with diazotrophic OTUs seemed to be related with water content and, therefore indirectly also with oxygen availability in the soil. Soils with higher water content contain more anoxic niches and shift in their redox potential (Fenchel et al., 2012). In turn, this leads to changes in the microbial community and the expression of genes related to anaerobic metabolisms (Ramírez-Flandes et al., 2019), which is also reflected in the higher proportion of anaerobic diazotrophs (cluster III and cluster I *Geobacter* reads) in grasslands with higher precipitation in the warmest season.

### Indications for Plants Influencing Diazotrophic Communities

Variation in perennial grass, grass-like, and forb cover most strongly correlated with variation in diazotrophic community composition. Some *Cyanobacteria* OTUs tended to decline in relative abundance with increasing forb and grass cover, which could be explained at some sites where light is more limited by dense plant cover. In contrast, *Deltaproteobacteria*-OTUs were positively correlated with forb cover and a higher soil C:N ratio. As this taxonomic group harbors anaerobic bacteria, we hypothesize that both variables might be linked to creating anoxic niches, potentially by increasing water retention, or respiration rates. One taxon assigned to *Clostridia* increased in relative abundance with increasing grass-like cover. As this OTU belongs to the genus *Cellulosilyticum*, it is most likely able to degrade cellulose (Miller et al., 2011) and could be associated with certain plants. Neither legume cover nor legume community composition showed any effect on the community composition of diazotrophs in our study, even though common root-nodulating rhizobia (*Azorhizobium*, *Bradyrhizobium*, *Mesorhizobium*, *Rhizobium*, and *Sinorhozobium*; Sprent et al., 2017) made up 21.7% of total *nifH* reads in our soils. Non-legume plants known to be associated with diazotrophs (Santi et al., 2013) were not present in our plant communities, and their respective diazotrophic genera were only detected with low abundance (*Azoarcus*, *Azospirillum*, *Azotobacter*, *Burkholderia*, *Gluconacetobacter*, *Herbaspirillum*, *Frankia*, and *Pseudomonas*; combined 1.6% of total *nifH* reads). Taken together, our vegetation data indicate that the abundance of perennial grasses, grass-like, and forbs influence diazotrophic communities. However, the observed correlations could also be explained by similar environmental conditions shaping both communities.

### Elevated Nutrient Levels Have a Limited Effect on the Diazotrophic Community

Most terrestrial ecosystems experience either direct or indirect nutrient inputs from anthropogenic activities (Mackenzie et al., 1998; Galloway et al., 2004; Bouwman et al., 2009), yet the effect of increased N or P availability on the composition of diazotrophic communities across a global range of grasslands has not been investigated. As P is known to enhance N_2_ fixation activity (Reed et al., 2007; Tang et al., 2017; Dynarski and Houlton, 2018; Wang et al., 2018; Smercina et al., 2019), we hypothesized that P addition would alleviate potential P limitation of diazotrophs and thus influence diazotrophic community composition as found in previous work (Che et al., 2018; Feng et al., 2018; Lin et al., 2018; Han et al., 2019). Although in parallel investigations of the same grassland soils, P addition induced a bacterial and fungal community shift (Leff et al., 2015), this was not the case for diazotrophs, which is in accordance with other studies (Wang et al., 2018; Hu et al., 2019). This indicates that different functional groups might respond differently to P addition and that diazotrophic communities in our grasslands are not strongly P-limited.

The effect of N addition was context-dependent, only influencing the diazotrophic community in two North American sites. This contrasts with parallel investigations at these sites, where a clear community shift was observed for bacteria, archaea, and fungi due to N addition (Leff et al., 2015). At the two sites that exhibited a diazotrophic community shift due to N, the relative read abundance of two *Cyanobacteria* OTUs decreased significantly, which were assigned to heterocystous *Cyanobacteria* genera (*Calothrix*, *Nostoc*). This indicates that this group might be specialized in fixing N_2_ and lost their competitive advantage under N fertilization. Alternatively, N fertilization could have promoted plant growth, decreasing light availability for cyanobacterial growth. This is consistent with cyanobacterial OTUs being negatively correlated with plant cover in our study. *Proteobacteria* OTUs at the two sites responded differently to N addition, with some declining and some increasing significantly, similar to the varying correlations between *Proteobacteria* OTUs and environmental factors seen in our study. As only a few OTUs significantly changed in relative abundance in response to N addition, we hypothesize that, relative to all other soil bacteria, the diazotrophic guild is more resilient to short-term changes in N availability. It remains to be seen whether shifts in community composition will occur after more prolonged exposure to chronically elevated nutrient supply. Additionally, investigating the effect of N and P addition on the quantitative abundance of diazotrophic communities and on other functional groups related to the N cycle is needed to increase our knowledge of how much anthropogenic influence will affect N cycle processes in grassland soils.

## Conclusion

In this study, we show that the diazotrophic community composition in globally distributed grassland soils is diverse and heterogeneous across sampling sites. We attribute this site effect mainly to a wide range of climatic and edaphic conditions selecting for a specific community, but likely in combination with processes constrained by spatial distance. To the best of our knowledge, this is the first study correlating seasonal climatic variables, vegetation cover, and spatial distance with diazotrophic beta diversity on a global scale. Best predictors for the diazotrophic OTU-based community composition seem to be related to water availability and the presence of certain perennial plant types. A genus-level-based analysis to reduce compositional heterogeneity across grasslands supported the effect of soil water availability but also extended the identified influencing factors to edaphic variables, like pH and soil cations. Our analysis further suggests that in the short-term, diazotrophic communities across grasslands are mainly resilient with slight community shifts after 4 years of chronic N addition, although the long-term impact of elevated nutrient supply remains to be investigated.

## Data Availability Statement

The datasets presented in this study can be found in online repositories. The names of the repository/repositories and accession number(s) can be found at: https://www.ncbi.nlm.nih.gov/, PRJNA777635.

## Author Contributions

DW and RA framed this research. EB and ES were coordinating the Nutrient Network globally and provided metadata. EB, BF, AM, RM, AR, MS, and ES were involved coordinating sample and data collection at single grassland sites. MN performed lab work and wrote the original draft with input from DW and RA. MN and RA analyzed the data. All authors contributed to the article and approved the submitted version.

## Funding

This research was financially supported by the Austrian Science Fund (FWF) [P25700-B20 to DW] and by the Austrian Science Fund (FWF) DK+ program ‘Microbial Nitrogen Cycling’ [W1257-B20]. MN was supported by a DOC fellowship (24388) from the Austrian Academy of Sciences (ÖAW). RA was supported by BC CAS, ISB & SoWa RI (MEYS; projects LM2015075, EF16_013/0001782-SoWa Ecosystems 315 Research). This work was generated using data from the Nutrient Network (http://www.nutnet.org) experiment, funded at the site-scale by individual researchers. Coordination and data management have been supported by funding to EB and ES from the National Science Foundation Research Coordination Network (NSF-DEB-1042132) and Long Term Ecological Research (NSF-DEB-1234162 and NSF-DEB-1831944 to Cedar Creek LTER) programs, and the Institute on the Environment (DG-0001-13).

## Conflict of Interest

The authors declare that the research was conducted in the absence of any commercial or financial relationships that could be construed as a potential conflict of interest.

## Publisher’s Note

All claims expressed in this article are solely those of the authors and do not necessarily represent those of their affiliated organizations, or those of the publisher, the editors and the reviewers. Any product that may be evaluated in this article, or claim that may be made by its manufacturer, is not guaranteed or endorsed by the publisher.

## References

[ref1] Altschul S. F. Madden T. L. Schäffer A. A. Zhang J. Zhang Z. Miller W.. (1997). Gapped BLAST and PSI-BLAST: a new generation of protein database search programs. Nucleic Acids Res. 25, 3389–3402. doi: 10.1093/nar/25.17.3389, PMID: 9254694 PMC146917

[ref2] Ando S. Goto M. Meunchang S. Thongra-ar P. Fujiwara T. Hayashi H.. (2005). Detection of *nifH* sequences in sugarcane (*Saccharum officinarum* L.) and pineapple (*Ananas comosus* [L.] Merr.). Soil Sci. Plant Nutr. 51, 303–308. doi: 10.1111/j.1747-0765.2005.tb00034.x

[ref3] Angel R. Nepel M. Panhölzl C. Schmidt H. Herbold C. W. Eichorst S. A.. (2018). Evaluation of primers targeting the diazotroph functional gene and development of NifMAP – a bioinformatics pipeline for analyzing *nifH* amplicon data. Front. Microbiol. 9:703. doi: 10.3389/fmicb.2018.00703, PMID: 29760683 PMC5936773

[ref4] Bates D. Mächler M. Bolker B. M. Walker S. C. (2015). Fitting linear mixed-effects models using lme4. J. Stat. Softw. 67, 1–48. doi: 10.18637/jss.v067.i01

[ref5] Benjamini Y. Hochberg Y. (1995). Controlling the false discovery rate: a practical and powerful approach to multiple testing. J. R. Stat. Soc. Ser. B 57, 289–300. doi: 10.1111/j.2517-6161.1995.tb02031.x

[ref6] Bennett E. M. Carpenter S. R. Caraco N. F. (2001). Human impact on erodable phosphorus and eutrophication: a global perspective. Bioscience 51, 227–234. doi: 10.1641/0006-3568(2001)051[0227:HIOEPA]2.0.CO;2

[ref7] Borer E. T. Harpole W. S. Adler P. B. Lind E. M. Orrock J. L. Seabloom E. W.. (2013). Finding generality in ecology: a model for globally distributed experiments. Methods Ecol. Evol. 5, 65–73. doi: 10.1111/2041-210X.12125

[ref8] Bouwman A. F. Beusen A. H. W. Billen G. (2009). Human alteration of the global nitrogen and phosphorus soil balances for the period 1970–2050. Glob. Biogeochem. Cycles 23:GB0A04. doi: 10.1029/2009GB003576

[ref9] Boyd E. S. Peters J. W. (2013). New insights into the evolutionary history of biological nitrogen fixation. Front. Microbiol. 4:201. doi: 10.3389/fmicb.2013.00201, PMID: 23935594 PMC3733012

[ref10] Carvalho T. L. G. Balsemão-Pires E. Saraiva R. M. Ferreira P. C. G. Hemerly A. S. (2014). Nitrogen signalling in plant interactions with associative and endophytic diazotrophic bacteria. J. Exp. Bot. 65, 5631–5642. doi: 10.1093/jxb/eru319, PMID: 25114015

[ref11] Che R. Deng Y. Wang F. Wang W. Xu Z. Hao Y.. (2018). Autotrophic and symbiotic diazotrophs dominate nitrogen-fixing communities in Tibetan grassland soils. Sci. Total Environ. 639, 997–1006. doi: 10.1016/j.scitotenv.2018.05.238, PMID: 29929338

[ref12] Chu H. Neufeld J. D. Walker V. K. Grogan P. (2011). The influence of vegetation type on the dominant soil bacteria, archaea, and fungi in a low arctic tundra landscape. Soil Sci. Soc. Am. J. 75, 1756–1765. doi: 10.2136/sssaj2011.0057

[ref13] Cleveland C. C. Townsend A. R. Schimel D. S. Fisher H. Hedin L. O. Perakis S.. (1999). Global patterns of terrestrial biological nitrogen (N_2_) fixation in natural ecosystems. Glob. Biogeochem. Cycles 13, 623–645. doi: 10.1029/1999GB900014

[ref14] Davey K. R. (1989). A predictive model for combined temperature and water activity on microbial growth during the growth phase. J. Appl. Bacteriol. 67, 483–488. doi: 10.1111/j.1365-2672.1989.tb02519.x, PMID: 2592289

[ref15] De Vries F. T. Shade A. (2013). Controls on soil microbial community stability under climate change. Front. Microbiol. 4:265. doi: 10.3389/fmicb.2013.00265, PMID: 24032030 PMC3768296

[ref16] Dynarski K. A. Houlton B. Z. (2018). Nutrient limitation of terrestrial free-living nitrogen fixation. New Phytol. 217, 1050–1061. doi: 10.1111/nph.14905, PMID: 29165820

[ref17] Erisman J. W. Galloway J. N. Seitzinger S. Bleeker A. Dise N. B. Roxana Petrescu A. M.. (2013). Consequences of human modification of the global nitrogen cycle. Philos. Trans. R. Soc. B Biol. Sci. 368:20130116. doi: 10.1098/rstb.2013.0116, PMID: 23713116 PMC3682738

[ref18] Fay P. A. Prober S. M. Harpole W. S. Knops J. M. H. Bakker J. D. Borer E. T.. (2015). Grassland productivity limited by multiple nutrients. Nat. Plants 1:15080. doi: 10.1038/nplants.2015.80, PMID: 27250253

[ref19] Fenchel T. King G. M. Blackburn T. H. (2012). “Chapter 6: Biogeochemical cycling in soils,” in Bacterial Biogeochemistry. eds. Fenchel T. King G. M. Blackburn T. H. (Boston: Academic Press), 89–120.

[ref20] Feng M. Adams J. M. Fan K. Shi Y. Sun R. Wang D.. (2018). Long-term fertilization influences community assembly processes of soil diazotrophs. Soil Biol. Biochem. 126, 151–158. doi: 10.1016/j.soilbio.2018.08.021

[ref21] Frank I. E. Turk-Kubo K. A. Zehr J. P. (2016). Rapid annotation of *nifH* gene sequences using classification and regression trees facilitates environmental functional gene analysis. Environ. Microbiol. Rep. 8, 905–916. doi: 10.1111/1758-2229.12455, PMID: 27557869

[ref22] Gaby J. C. Buckley D. H. (2011). A global census of nitrogenase diversity. Environ. Microbiol. 13, 1790–1799. doi: 10.1111/j.1462-2920.2011.02488.x, PMID: 21535343

[ref23] Galloway J. N. Dentener F. J. Capone D. G. Boyer E. W. Howarth R. W. Seitzinger S. P.. (2004). Nitrogen cycles: past, present, and future. Biogeochemistry 70, 153–226. doi: 10.1007/s10533-004-0370-0

[ref24] Goslee S. C. Urban D. L. (2007). The ecodist package for dissimilarity-based analysis of ecological data. J. Stat. Softw. 22, 1–19. doi: 10.18637/jss.v022.i07

[ref25] Graham P. H. Draeger K. J. Ferrey M. L. Conroy M. J. Hammer B. E. Martinez E.. (1994). Acid pH tolerance in strains of *Rhizobium* and *Bradyrhizobium*, and initial studies on the basis for acid tolerance of *Rhizobium trpici* UMR1899. Can. J. Microbiol. 40, 198–207. doi: 10.1139/m94-033

[ref26] Han L.-L. Wang Q. Shen J.-P. Di H. J. Wang J.-T. Wei W.-X.. (2019). Multiple factors drive the abundance and diversity of the diazotrophic community in typical farmland soils of China. FEMS Microbiol. Ecol. 95:fiz113. doi: 10.1093/femsec/fiz113, PMID: 31295349

[ref27] Herbold C. W. Pelikan C. Kuzyk O. Hausmann B. Angel R. Berry D.. (2015). A flexible and economical barcoding approach for highly multiplexed amplicon sequencing of diverse target genes. Front. Microbiol. 6:731. doi: 10.3389/fmicb.2015.00731, PMID: 26236305 PMC4503924

[ref28] Hijmans R. J. (2019). Geosphere: Spherical Trigonometry. Available at: https://cran.r-project.org/package=geosphere (accessed on 20 September 2021).

[ref29] Hijmans R. J. Cameron S. E. Parra J. L. Jones P. G. Jarvis A. (2005). Very high resolution interpolated climate surfaces for global land areas. Int. J. Climatol. 25, 1965–1978. doi: 10.1002/joc.1276

[ref30] Hu X. Liu J. Wei D. Zhou B. Chen X. Jin J.. (2019). Long-term application of nitrogen, not phosphate or potassium, significantly alters the diazotrophic community compositions and structures in a Mollisol in Northeast China. Res. Microbiol. 170, 147–155. doi: 10.1016/j.resmic.2019.02.002 30817988

[ref31] IPCC (2019). “Climate change and land: an IPCC special report on climate change, desertification, land degradation, sustainable land management, food security, and greenhouse gas fluxes in terrestrial ecosystems,” eds. Shukla P. R. Skea J. Buendia E. C. Masson-Delmotte V. Pörtner H.-O. Roberts D. C.. (Intergovernmental Panel on Climate Change (IPCC)), (in press).

[ref32] Kimber R. L. Bagshaw H. Smith K. Buchanan D. M. Coker V. S. Cavet J. S.. (2020). Biomineralization of Cu_2_S nanoparticles by *Geobacter sulfurreducens*. Appl. Environ. Microbiol. 86, e00967–e001020. doi: 10.1128/AEM.00967-20, PMID: 32680873 PMC7480366

[ref33] Kramer P. J. (1983). “Soil and water,” in Water Relations of Plants. ed. Kramer P. J. (New York: Academic Press), 57–83.

[ref34] Ladomersky E. Petris M. J. (2015). Copper tolerance and virulence in bacteria. Metallomics 7, 957–964. doi: 10.1039/c4mt00327f.Copper, PMID: 25652326 PMC4464932

[ref35] Lahti L. Shetty S. (2017). Tools for Microbiome Analysis in R. Available at: https://microbiome.github.io/tutorials/ (accessed on 1 February 2022).

[ref36] Leff J. W. Jones S. E. Prober S. M. Barberán A. Borer E. T. Firn J. L.. (2015). Consistent responses of soil microbial communities to elevated nutrient inputs in grasslands across the globe. Proc. Natl. Acad. Sci. U. S. A. 112, 10967–10972. doi: 10.1073/pnas.1508382112, PMID: 26283343 PMC4568213

[ref37] Lichstein J. W. (2007). Multiple regression on distance matrices: a multivariate spatial analysis tool. Plant Ecol. 188, 117–131. doi: 10.1007/s11258-006-9126-3

[ref38] Lin Y. Ye G. Liu D. Ledgard S. Luo J. Fan J.. (2018). Long-term application of lime or pig manure rather than plant residues suppressed diazotroph abundance and diversity and altered community structure in an acidic Ultisol. Soil Biol. Biochem. 123, 218–228. doi: 10.1016/j.soilbio.2018.05.018

[ref39] Luo G. Shi Z. Wang H. Wang G. (2012). *Skermanella stibiiresistens* sp. nov., a highly antimony-resistant bacterium isolated from coal-mining soil, and emended description of the genus *Skermanella*. Int. J. Syst. Evol. Microbiol. 62, 1271–1276. doi: 10.1099/ijs.0.033746-0, PMID: 21784960

[ref40] Mackenzie F. T. Ver L. M. Lerman A. (1998). “Coupled biogeochemical cycles of carbon, nitrogen, phosphorus and sulfur in the land-ocean-atmosphere system,” in Asian Change in the Context of Global Climate Change. eds. Galloway J. N. Melillo J. M. (New York: Cambridge University Press), 42–100.

[ref41] Martin B. D. Witten D. Willis A. D. (2020). Modeling microbial abundances and dysbiosis with beta-binomial regression. Ann. Appl. Stat. 14, 94–115. doi: 10.1214/19-AOAS1283, PMID: 32983313 PMC7514055

[ref42] Martiny J. B. H. Eisen J. A. Penn K. Allison S. D. Horner-Devine M. C. (2011). Drivers of bacterial β-diversity depend on spatial scale. Proc. Natl. Acad. Sci. U. S. A. 108, 7850–7854. doi: 10.1073/pnas.1016308108, PMID: 21518859 PMC3093525

[ref43] McArdle B. H. Anderson M. J. Ecology S. Jan N. (2001). Fitting multivariate models to community data: a comment on distance-based redundancy analysis. Ecology 82, 290–297. doi: 10.1890/0012-9658(2001)082[0290:FMMTCD]2.0.CO;2

[ref44] McMurdie P. J. Holmes S. (2013). phyloseq: an R package for reproducible interactive analysis and graphics of microbiome census data. PLoS One 8:e61217. doi: 10.1371/journal.pone.0061217, PMID: 23630581 PMC3632530

[ref45] Miller D. A. Suen G. Bruce D. Copeland A. Cheng J. F. Detter C.. (2011). Complete genome sequence of the cellulose-degrading bacterium *Cellulosilyticum lentocellum*. J. Bacteriol. 193, 2357–2358. doi: 10.1128/JB.00239-11, PMID: 21398547 PMC3133088

[ref46] Nayak S. Prasanna R. (2007). Soil pH and its role in cyanobacterial abundance and diversity in rice field soils. Appl. Ecol. Environ. Res. 5, 103–113. doi: 10.15666/aeer/0502_103113

[ref47] Oksanen J. Blanchet F. G. Friendly M. Kindt R. Legendre P. McGlinn D.. (2019). Vegan: Community Ecology Package. Available at: https://cran.r-project.org/package=vegan (accessed on 6 July 2019).

[ref48] Ozawa T. Imai Y. Sukiman H. I. Karsono H. Ariani D. Saono S. (1999). Low ph and aluminum tolerance of *Bradyrhizobium* strains isolated from acid soils in Indonesia. Soil Sci. Plant Nutr. 45, 987–992. doi: 10.1080/00380768.1999.10414349

[ref49] Pandey K. D. Shukla P. N. Giri D. D. Kashyap A. K. (2005). *Cyanobacteria* in alkaline soil and the effect of cyanobacteria inoculation with pyrite amendments on their reclamation. Biol. Fertil. Soils 41, 451–457. doi: 10.1007/s00374-005-0846-7

[ref50] Prober S. M. Leff J. W. Bates S. T. Borer E. T. Firn J. Harpole W. S.. (2014). Plant diversity predicts beta but not alpha diversity of soil microbes across grasslands worldwide. Ecol. Lett. 18, 85–95. doi: 10.1111/ele.12381 25430889

[ref51] Pruitt K. D. Tatusova T. Maglott D. R. (2007). NCBI reference sequences (RefSeq): a curated non-redundant sequence database of genomes, transcripts and proteins. Nucleic Acids Res. 35, D61–D65. doi: 10.1093/nar/gkl842, PMID: 17130148 PMC1716718

[ref52] R Core Team (2018). R: A Language and Environment for Statistical Computing. Available at: https://www.r-project.org/ (accessed on 6 July 2019).

[ref53] Ramírez-Flandes S. González B. Ulloa O. (2019). Redox traits characterize the organization of global microbial communities. Proc. Natl. Acad. Sci. U. S. A. 116, 3630–3635. doi: 10.1073/pnas.1817554116, PMID: 30808753 PMC6397516

[ref54] Rawls W. J. Pachepsky Y. A. Ritchie J. C. Sobecki T. M. Bloodworth H. (2003). Effect of soil organic carbon on soil water retention. Geoderma 116, 61–76. doi: 10.1016/S0016-7061(03)00094-6

[ref55] Raymond J. Siefert J. L. Staples C. R. Blankenship R. E. (2004). The natural history of nitrogen fixation. Mol. Biol. Evol. 21, 541–554. doi: 10.1093/molbev/msh047 14694078

[ref56] Reed S. C. Cleveland C. C. Townsend A. R. (2011). Functional ecology of free-living nitrogen fixation: a contemporary perspective. Annu. Rev. Ecol. Evol. Syst. 42, 489–512. doi: 10.1146/annurev-ecolsys-102710-145034

[ref57] Reed S. C. Seastedt T. R. Mann C. M. Suding K. N. Townsend A. R. Cherwin K. L. (2007). Phosphorus fertilization stimulates nitrogen fixation and increases inorganic nitrogen concentrations in a restored prairie. Appl. Soil Ecol. 36, 238–242. doi: 10.1016/j.apsoil.2007.02.002

[ref58] Reinhold-Hurek B. Hurek T. (1998). Life in grasses: diazotrophic endophytes. Trends Microbiol. 6, 139–144. doi: 10.1016/S0966-842X(98)01229-3, PMID: 9587190

[ref59] Rillig M. C. Ryo M. Lehmann A. Aguilar-Trigueros C. A. Buchert S. Wulf A.. (2019). The role of multiple global change factors in driving soil functions and microbial biodiversity. Science 366, 886–890. doi: 10.1126/science.aay2832, PMID: 31727838 PMC6941939

[ref60] Rinkel B. E. Manoylov K. M. (2014). *Calothrix*: an evaluation of fresh water species in United States rivers and streams, their distribution and preliminary ecological findings source. Proc. Acad. Nat. Sci. Philadelphia 163, 43–59. doi: 10.1635/053.163.0108

[ref61] Santi C. Bogusz D. Franche C. (2013). Biological nitrogen fixation in non-legume plants. Ann. Bot. 111, 743–767. doi: 10.1093/aob/mct048, PMID: 23478942 PMC3631332

[ref62] Schauer R. Bienhold C. Ramette A. Harder J. (2010). Bacterial diversity and biogeography in deep-sea surface sediments of the South Atlantic Ocean. ISME J. 4, 159–170. doi: 10.1038/ismej.2009.106, PMID: 19829317

[ref63] Schimel J. P. (2018). Life in dry soils: effects of drought on soil microbial communities and processes. Annu. Rev. Ecol. Evol. Syst. 49, 409–432. doi: 10.1146/annurev-ecolsys-110617-062614

[ref64] Shen C. Xiong J. Zhang H. Feng Y. Lin X. Li X.. (2013). Soil pH drives the spatial distribution of bacterial communities along elevation on Changbai Mountain. Soil Biol. Biochem. 57, 204–211. doi: 10.1016/j.soilbio.2012.07.013

[ref65] Smercina D. N. Evans S. E. Friesen M. L. Tiemann L. K. (2019). To fix or not to fix: controls on free-living nitrogen fixation in the rhizosphere. Appl. Environ. Microbiol. 85, e02546–e02618. doi: 10.1128/AEM.02546-18, PMID: 30658971 PMC6414387

[ref66] Sprent J. I. Ardley J. James E. K. (2017). Biogeography of nodulated legumes and their nitrogen-fixing symbionts. New Phytol. 215, 40–56. doi: 10.1111/nph.14474, PMID: 28211601

[ref67] Tang Y. Zhang M. Chen A. Zhang W. Wei W. Sheng R. (2017). Impact of fertilization regimes on diazotroph community compositions and N_2_-fixation activity in paddy soil. Agric. Ecosyst. Environ. 247, 1–8. doi: 10.1016/j.agee.2017.06.009

[ref68] Telford R. J. Vandvik V. Birks H. J. B. (2006). Dispersal limitations matter for microbial morphospecies. Science 312:1015. doi: 10.1126/science.1125669, PMID: 16709777

[ref69] Tognetti P. M. Prober S. M. Báez S. Chaneton E. J. Firn J. Risch A. C.. (2021). Negative effects of nitrogen override positive effects of phosphorus on grassland legumes worldwide. Proc. Natl. Acad. Sci. U. S. A. 118:e2023718118. doi: 10.1073/pnas.2023718118, PMID: 34260386 PMC8285913

[ref70] Trabucco A. Zomer R. J. (2018). Global Aridity Index and Potential Evapo-Transpiration (et0) Climate Database v2. CGIAR Consort. Spat. Inf. Available at: https://cgiarcsi.community (accessed on 20 October 2020).

[ref71] Trevors J. T. Cotter C. M. (1990). Copper toxicity and uptake in microorganisms. J. Ind. Microbiol. 6, 77–84. doi: 10.1007/BF01576426

[ref72] Van Der Gucht K. Cottenie K. Muylaert K. Vloemans N. Cousin S. Declerck S.. (2007). The power of species sorting: local factors drive bacterial community composition over a wide range of spatial scales. Proc. Natl. Acad. Sci. U. S. A. 104, 20404–20409. doi: 10.1073/pnas.0707200104, PMID: 18077371 PMC2154443

[ref73] Vitousek P. M. Cassman K. Cleveland C. Crews T. Field C. B. Grimm N. B.. (2002). Towards an ecological understanding of biological nitrogen fixation. Biogeochemistry 57–58, 1–45. doi: 10.1023/A:1015798428743

[ref74] Vitousek P. M. Howarth R. W. (1991). Nitrogen limitation on land and in the sea: how can it occur? Biogeochemistry 13, 87–115. doi: 10.1007/BF00002772

[ref75] Wang Y. Li C. Kou Y. Wang J. Tu B. Li H.. (2017a). Soil pH is a major driver of soil diazotrophic community assembly in Qinghai-Tibet alpine meadows. Soil Biol. Biochem. 115, 547–555. doi: 10.1016/j.soilbio.2017.09.024

[ref76] Wang Q. Wang J. Li Y. Chen D. Ao J. Zhou W.. (2018). Influence of nitrogen and phosphorus additions on N_2_-fixation activity, abundance, and composition of diazotrophic communities in a Chinese fir plantation. Sci. Total Environ. 619–620, 1530–1537. doi: 10.1016/j.scitotenv.2017.10.064 29129329

[ref77] Wang J. Wu Y. Jiang H. Li C. Dong H. Wu Q.. (2008). High beta diversity of bacteria in the shallow terrestrial subsurface. Environ. Microbiol. 10, 2537–2549. doi: 10.1111/j.1462-2920.2008.01678.x, PMID: 18833648

[ref78] Wang C. Zheng M. Song W. Wen S. Wang B. Zhu C.. (2017b). Impact of 25 years of inorganic fertilization on diazotrophic abundance and community structure in an acidic soil in southern China. Soil Biol. Biochem. 113, 240–249. doi: 10.1016/j.soilbio.2017.06.019

[ref79] White R. Murray S. Rohweder M. (2000). Pilot Analysis of Global Ecosystems: Grassland Ecosystems. Washington, DC: World Resources Institute.

[ref80] Wickham H. (2016). ggplot2: Elegant Graphics for Data Analysis. Springer-Verlag New York Available at: https://ggplot2.tidyverse.org (accessed on 6 March 2021).

[ref81] Zehr J. Jenkins B. Short S. Steward G. (2003). Nitrogenase gene diversity and microbial community structure: a cross-system comparison. Environ. Microbiol. 5, 539–554. doi: 10.1046/j.1462-2920.2003.00451.x, PMID: 12823187

[ref82] Zheng H. Dietrich C. Radek R. Brune A. (2015). *Endomicrobium proavitum*, the first isolate of *Endomicrobia* class. nov. (phylum *Elusimicrobia*)—an ultramicrobacterium with an unusual cell cycle that fixes nitrogen with a group IV nitrogenase. Environ. Microbiol. 18, 191–204. doi: 10.1111/1462-2920.12960, PMID: 26119974

