# Supplementary material for: Global Grassland Diazotrophic Communities Are Structured by Combined Abiotic, Biotic, and Spatial Distance Factors but Resilient to Fertilization
Source: Front Microbiol. 2022 Mar 28;13:821030. doi: 10.3389/fmicb.2022.821030 (PMC8996192; doi:10.3389/fmicb.2022.821030)
Supplement: Supplementary file 1 [file Data_Sheet_1.PDF]

Supplementary Material

Supplementary Figures

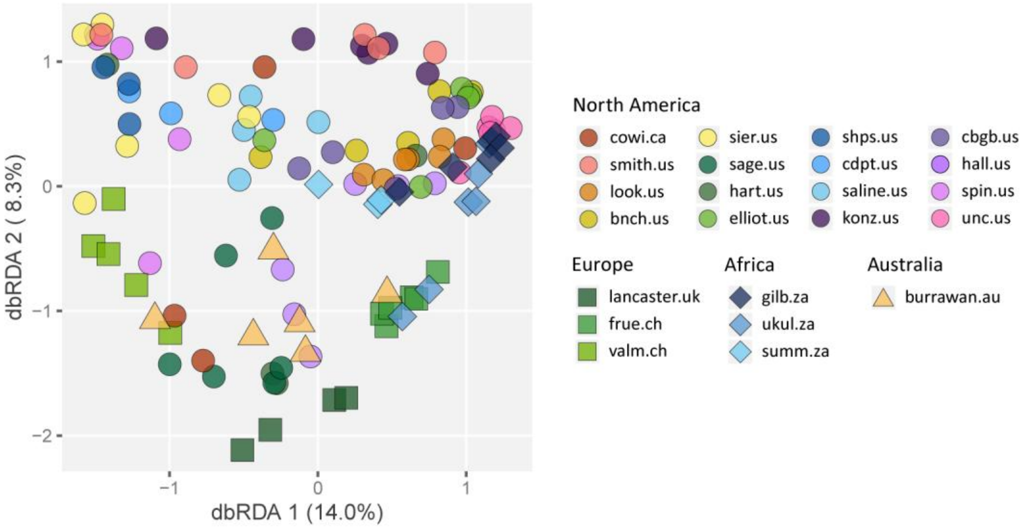

Figure S1: Distance-based redundancy analysis (dbRDA) displaying the effect of study sites on the Bray-Curtis dissimilarity of diazotrophic communities at the genus level. Each color represents one collection site and shapes denote the respective continent.

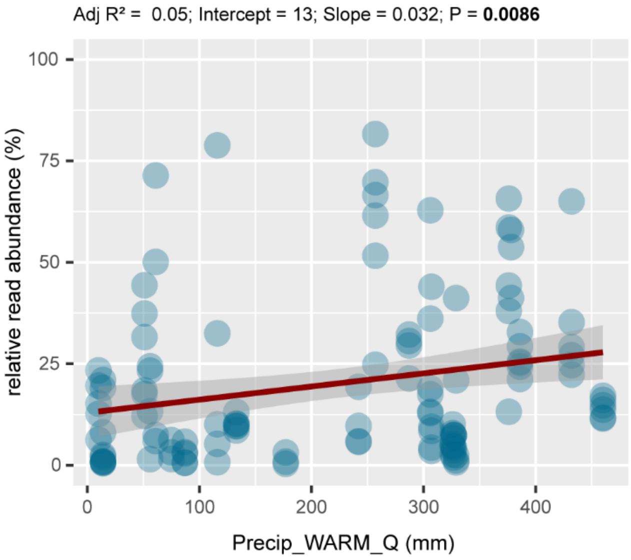

Figure S2: Linear model of alleged anoxic diazotrophs and the environmental variable Precip_WARM_Q. The x-axis denotes the mean precipitation in the warmest quarter (mm) at study sites, and the y-axis shows the relative abundance of cluster III and anaerobic *Geobacter* cluster I reads, combined.

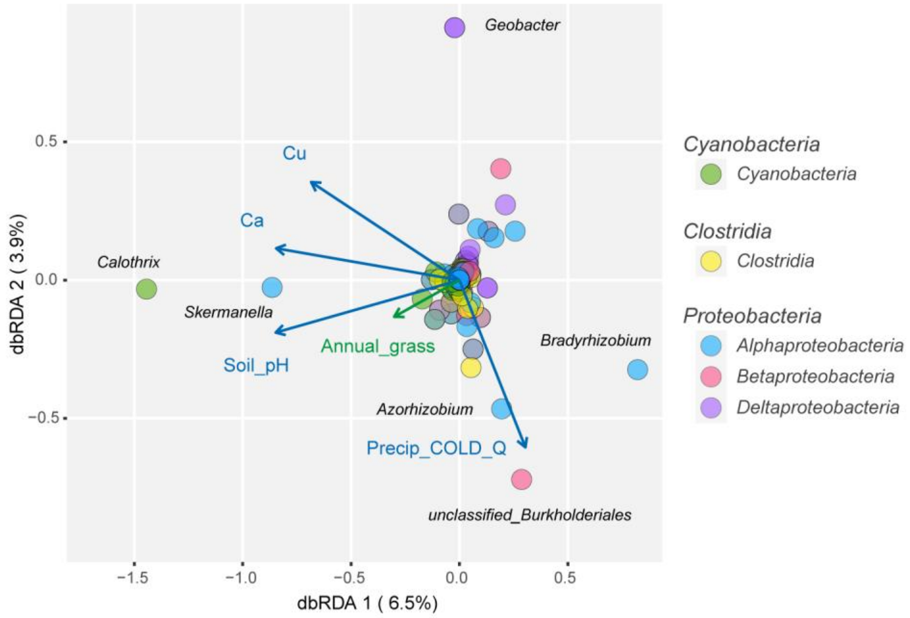

Figure S3: Distance-based redundancy analysis (dbRDA) showing the variation of the diazotrophs of relevant taxonomic classes on genus level as a factor of different environmental parameters based on our best descriptive model. Four abiotic variables, soil pH, Ca, Cu, mean precipitation in the coldest quarter (Precip_COLD_Q), and one plant variable, annual grass cover, are depicted as blue or green vectors, respectively. Genera, significantly correlating with depicted environmental factors, are labeled. Colored circles denote genera assigned to the five most read-abundant taxonomic classes. Genera of other taxonomic classes are depicted in grey.

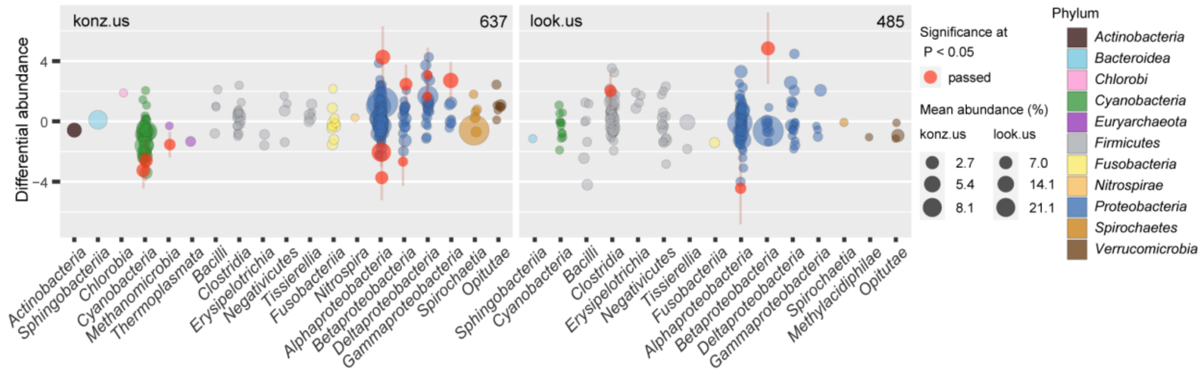

Figure S4: Differential abundance of OTUs between control plots and N fertilization of both significant sites konz.us and look.us, using corncob. The x-axis shows the classification of each OTU at the class level, whereas the y-axis shows the difference in the modeled relative abundance between treatments. Only OTUs present in at least 2.5% of overall samples were used for this analysis (number shown per site). Each circle denotes a single OTU, and its size is its average relative abundance across all samples. The red circles highlight OTUs significantly changing in relative read abundance. Otherwise, the circle color depicts the assigned taxonomic phylum.

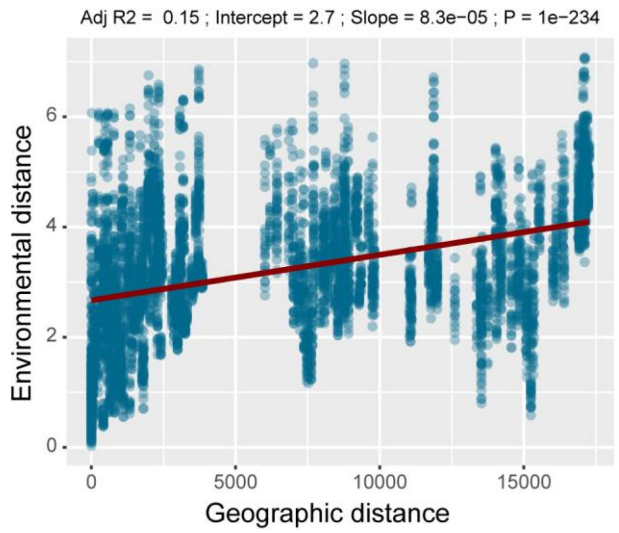

Figure S5: Linear model showing the correlation between the geographic and environmental distance matrices. Every circle represents the spatial and the environmental distance between two samples. The x-axis depicts the linear geographical distance (in km) between two samples. The y-axis represents the environmental dissimilarity between two samples, based on a Euclidean distance matrix including all six normalized abiotic and biotic variables correlating most with the diazotrophic community composition (Precip_WARM_Q, Temp_WET_Q, soil_C:N, Perennial grass, grass-like and forb cover).

Supplementary Tables

Table S1: Abbreviations of the studied grassland sites and their geographic locations. Selected environmental variables are listed in Table S5 and Table S8.

Site code	Site name	Region	Continent	Latitude	Longitude
bnch.us	Bunchgrass (Andrews LTER)	Intermountain_west	North America	44.277	-121.968
burrawan.au	Burrawan	Australia	Australia	-27.735	151.140
cbgb.us	Chichaqua Bottoms	Central	North America	41.785	-93.385
cdpt.us	Cedar Point Biological Station	Central	North America	41.200	-101.630
cowi.ca	Cowichan	Pacific_coast	North America	48.460	-123.380
elliot.us	Elliott Chaparral	Pacific_coast	North America	32.875	-117.052
frue.ch	Fruebuel	Europe	Europe	47.113	8.542
gilb.za	Mt Gilboa	Africa	Africa	-29.284	30.292
hall.us	Halls Prairie	Central	North America	36.872	-86.702
hart.us	Hart Mountain	Intermountain_west	North America	42.724	-119.498
konz.us	Konza LTER	Central	North America	39.071	-96.583
lancaster.uk	Lancaster	Europe	Europe	53.986	-2.628
look.us	Lookout (Andrews LTER)	Intermountain_west	North America	44.205	-122.128
sage.us	Sagehen Creek UCNRS	Intermountain_west	North America	39.430	-120.240
saline.us	Saline Experimental Range	Central	North America	39.050	-99.100
shps.us	Sheep Experimental Station	Intermountain_west	North America	44.243	-112.198
sier.us	Sierra Foothills REC	Pacific_coast	North America	39.236	-121.284
smith.us	Smith Prairie	Pacific_coast	North America	48.207	-122.625
spin.us	Spindletop	Central	North America	38.136	-84.501
summ.za	Summerveld	Africa	Africa	-29.812	30.716
ukul.za	Ukulinga	Africa	Africa	-29.670	30.400
unc.us	Duke Forest	Atlantic_coast	North America	36.008	-79.020
valm.ch	Val Mustair	Europe	Europe	46.631	10.372

Table S2: Information on removed reads and OTUs during sequence data processing. Details on the used bioinformatics pipeline NifMAP can be retrieved from Angel *et al.* (2018).

Process step	Input	Remaining	Removed
	No. of reads		%
Quality filtering and trimming of raw reads	2,723,627	2,296,354	15.69
Hmmscreen (nucleotide level) and chimera check of only unique and non-singleton reads	120,686	111,637	7.50
	No. of OTUs		%
Hmmscreen (protein level) and chimera check after OTU clustering	12,664	6,857	45.85
Removing specific samples, e.g. < 500 reads	6,857	6,826	0.45
Remaining dataset for analyses			
No. of reads		667,896	
No. of OTUs		6,826	

Table S3: Mantel Test statistics for correlations between diazotrophic beta diversity at OTU level and abiotic environmental variables. P-values are corrected for multiple testing, according to Benjamini-Hochberg.

Variable	Spearman's ρ	P (adjusted)
geographic distance	0.3868	**0.002**
elevation	0.2155	**0.002**
MAT	0.2405	**0.002**
MAX_TEMP	0.1650	**0.002**
MIN_TEMP	0.1190	**0.002**
Temp_WET_Q	0.3409	**0.002**
Temp_DRY_Q	0.0320	0.187
Temp_WARM_Q	0.2271	**0.002**
Temp_COLD_Q	0.1661	**0.002**
MAP	0.1402	**0.002**
Precip_WET_M	0.1583	**0.002**
Precip_DRY_M	0.0582	**0.038**
Precip_WET_Q	0.1626	**0.002**
Precip_DRY_Q	0.0768	**0.006**
Precip_WARM_Q	0.2561	**0.002**
Precip_COLD_Q	0.2268	**0.002**
Aridity_Index	0.1151	**0.004**
PercentSand	0.2462	**0.002**
PercentClay	0.2911	**0.002**
pct_C	0.1589	**0.002**
pct_N	0.1700	**0.002**
ppm_P	0.0257	0.284
ppm_K	-0.0790	0.953
ppm_Ca	0.1096	**0.003**
ppm_Mg	0.0489	0.187
ppm_S	0.0424	0.133
ppm_Na	0.0002	0.529
ppm_Zn	0.0138	0.427
ppm_Mn	0.0968	**0.006**
ppm_Fe	-0.0062	0.590
ppm_Cu	0.0582	0.102
ppm_B	0.0006	0.529
soil_CN	0.2272	**0.002**
soil_CP	0.1250	**0.002**
soil_NP	0.1218	**0.004**
soil_pH	0.0435	0.153

mean annual temperature (MAT)
mean annual precipitation (MAP)
month (M)
quarter (Q)

Table S4: Mantel Test statistics for correlations between diazotrophic beta diversity at OTU level and biotic vegetation variables. P-values are corrected for multiple testing, according to Benjamini-Hochberg.

Variable	Spearmen's *ρ*	P (adjusted)
**Cover types**		
Annual_forb	-0.0719	0.986
Annual_grass	-0.0081	0.966
Annual_legume	-0.0333	0.986
Biennial_forb	-0.0419	0.986
Bryophyte	0.0788	0.153
Lichen	0.0767	0.177
Perennial_forb	0.1226	**0.006**
Perennial _grass-like	0.1515	**0.009**
Perennial _grass	0.2375	**0.006**
Perennial _legume	0.0503	0.232
Perennial _woody	-0.0987	0.986
**plant communities**		
total plant community	0.0820	**0.042**
Perennial _grass	0.2731	**0.001**
Perennial _grass-like	0.1887	**0.001**
Perennial_forb	0.2571	**0.001**
Perennial _legume	0.0558	0.085

Table S5: Average environmental conditions per study site. Listed are variables of the best model describing diazotrophic beta diversity at OTU level.

site_code	Percent sand (%)		Percent clay (%)		Soil_CN (ratio)		Temp_ WET_Q (°C)	Precip_ WARM_Q (mm)	Perennial_ forb_cover (%)		Perennial_ grass-like_cover (%)		Perennial_ grass_cover (%)	
	mean	sd	mean	sd	mean	sd	mean	mean	mean	sd	mean	sd	mean	sd
bnch.us	70.1	1.6	3.6	2.8	13.8	0.6	-1.1	116	60.0	45.2	44.0	16.7	17.2	6.8
burrawan.au	83.5	*NA*	9.3	*NA*	15.6	1.5	24.4	257	3.2	1.0	6.8	6.6	76.8	20.7
cbgb.us	89.0	1.4	4.8	2.8	9.9	0.6	20.1	329	27.2	9.4	0	0	88.4	29.5
cdpt.us	66.6	8.6	9.5	2.8	11.3	0.4	19.7	177	3.9	2.3	24.7	5.5	26.5	8.2
cowi.ca	25.4	*NA*	31.3	*NA*	14.2	1.2	5.3	61	1.8	2.9	0	0	45.8	16.8
elliot.us	54.2	4.5	20.1	3.1	13.2	0.8	13.3	10	10.6	10.9	1.0	1.4	10.2	17.2
frue.ch	38.2	7.6	20.6	5.8	10.3	0.3	14.1	460	57.0	15.5	0	0	86.7	15.4
gilb.za	*NA*	*NA*	*NA*	*NA*	17.7	0.8	16.9	432	35.0	8.9	16.2	20.2	131.8	34.3
hall.us	25.1	3.4	15.5	0.0	10.6	0.5	13.6	306	28.3	13.7	0	0	49.3	27.4
hart.us	48.0	1.4	27.5	5.7	12.0	0.6	10.0	56	1.0	0.7	0	0	10.8	5.7
konz.us	*NA*	*NA*	*NA*	*NA*	14.0	0.4	21.9	327	30.2	10.9	21.2	8.7	144.2	17.4
lancaster.uk	50.2	1.2	18.6	2.3	20.0	0.7	3.3	287	34.5	39.4	1.3	2.5	63.0	31.6
look.us	70.0	2.0	0.8	0.0	14.2	1.4	-1.4	133	50.0	19.7	22.5	16.1	21.0	10.9
sage.us	44.8	6.0	24.1	4.9	13.0	1.1	-2.2	51	33.9	14.8	7.2	8.0	8.0	7.4
saline.us	*NA*	*NA*	*NA*	*NA*	13.5	1.6	22.1	242	14.8	12.4	0.5	1.0	114.3	20.6
shps.us	52.0	5.7	11.8	4.2	12.2	1.0	15.0	75	6.0	0	0	0	19.3	4.9
sier.us	38.7	12.2	18.8	4.0	12.1	0.7	8.2	14	1.7	4.1	0	0	0	0
smith.us	78.0	3.5	6.8	3.5	13.1	0.2	5.0	87	18.5	8.7	0	0	22.0	7.1
spin.us	29.3	6.4	20.8	2.0	10.3	0.3	12.1	307	1.6	0.6	0	0	11.8	12.3
summ.za	*NA*	*NA*	*NA*	*NA*	19.3	0.9	20.3	378	54.7	9.5	3.7	0.6	121.0	13.1
ukul.za	18.3	3.9	44.6	3.0	16.5	0.5	21.3	386	57.5	13.7	0	0	99.2	18.7
unc.us	56.0	4.5	21.3	3.5	13.9	1.4	24.2	326	5.1	5.5	0	0	73.2	11.7
valm.ch	57.6	2.9	13.3	0.0	13.1	0.9	7.1	376	43.3	10.3	3.9	3.4	27.9	9.4

Table S6: Mantel Test statistics for correlations between diazotrophic beta diversity clustered at taxonomic genus level and abiotic environmental variables. P-values are corrected for multiple testing, according to Benjamini-Hochberg.

Variable	Spearmen's ρ	P (adjusted)
geographic distance	0.0130	0.322
elevation	0.0800	**0.018**
MAT	0.1003	**0.003**
MAX_TEMP	0.1446	**0.003**
MIN_TEMP	0.0814	**0.003**
Temp_WET_Q	0.1362	**0.003**
Temp_DRY_Q	0.0858	**0.014**
Temp_WARM_Q	0.1139	**0.003**
Temp_COLD_Q	0.0599	0.055
MAP	0.1166	**0.006**
Precip_WET_M	0.2165	**0.003**
Precip_DRY_M	0.0264	0.212
Precip_WET_Q	0.1960	**0.003**
Precip_DRY_Q	0.0324	0.178
Precip_WARM_Q	0.0998	**0.003**
Precip_COLD_Q	0.1596	**0.003**
PercentSand	0.0534	0.161
PercentClay	-0.0204	0.606
Aridity_Index	0.0977	**0.029**
pct_C	0.0444	0.175
pct_N	0.0638	0.094
ppm_P	0.0583	0.115
ppm_K	0.0818	0.064
ppm_Ca	0.2058	**0.003**
ppm_Mg	0.1425	**0.008**
ppm_S	0.0149	0.335
ppm_Na	-0.0051	0.537
ppm_Zn	0.0141	0.389
ppm_Mn	0.0621	**0.047**
ppm_Fe	0.0550	0.101
ppm_Cu	0.1604	**0.006**
ppm_B	0.1111	**0.023**
soil_CN	0.0240	0.275
soil_CP	-0.0119	0.635
soil_NP	0.0040	0.490
soil_pH	0.2228	**0.003**

mean annual temperature (MAT)
mean annual precipitation (MAP)
month (M)
quarter (Q)

Table S7: Mantel Test statistics for correlations between diazotrophic beta diversity of unamended treatment plots clustered at taxonomic genus level and biotic vegetation variables. P-values are corrected for multiple testing, according to Benjamini-Hochberg.

Variable	Spearmen's ρ	P (adjusted)
**Cover types**		
Annual_forb	0.1637	**0.007**
Annual_grass	0.2471	**0.007**
Annual_legume	0.1853	**0.007**
Biennial_forb	-0.0003	0.651
Bryophyte	0.0645	0.224
Lichen	0.0861	0.200
Perennial_forb	0.0517	0.200
Perennial_grass-like	0.0407	0.398
Perennial_grass	0.0466	0.200
Perennial_legume	0.0103	0.642
Perennial_woody	0.0105	0.642

Table S8: Average environmental conditions per study site. Listed are variables of the best model describing diazotrophic beta diversity at genus level.

site_code	Ca (ppm)		Cu (ppm)		soil_pH		Precip_ COLD_Q (mm)	Annual_ grass_cover (%)	
	mean	sd	mean	sd	mean	sd	mean	mean	sd
bnch.us	252.4	52.5	1.68	0.08	5.54	0.11	741	11.8	13.5
burrawan.au	666.0	367.3	2.47	0.45	5.55	0.51	109	1.7	2.1
cbgb.us	605.6	168.8	1.98	0.87	6.16	0.46	81	0	0
cdpt.us	1608.0	129.3	1.63	0.21	6.70	0.36	39	19.7	14.0
cowi.ca	2894.5	564.0	3.38	0.47	5.50	0.22	339	0	0
elliot.us	1497.4	253.1	1.38	0.13	5.74	0.15	167	120.0	16.6
frue.ch	1709.5	264.5	2.83	0.26	5.52	0.20	267	0	0
gilb.za	198.0	17.0	2.35	0.07	5.13	0.08	53	0	0
hall.us	800.3	87.7	1.75	0.27	5.17	0.14	326	0	0
hart.us	2927.5	769.8	4.08	0.10	7.25	0.10	67	0.4	0.3
konz.us	2609.0	213.7	2.88	0.69	6.65	0.21	73	0	0
lancaster.uk	1530.0	646.8	2.22	0.77	4.75	0.29	378	0	0
look.us	677.0	286.1	1.37	0.23	5.10	0.18	850	0	0
sage.us	3869.2	919.8	3.50	0.38	6.03	0.32	413	0	0
saline.us	5117.7	1793.3	4.80	0.87	7.97	0.40	45	0	0
shps.us	8063.7	695.0	4.87	0.25	8.20	0.00	61	1	0
sier.us	2231.0	574.4	4.92	3.22	6.00	0.09	477	103.2	12.7
smith.us	2343.2	910.1	1.68	0.26	6.20	0.42	208	0.9	1.2
spin.us	2520.8	299.0	1.96	0.15	6.40	0.14	259	0	0
summ.za	501.0	251.7	1.50	0.10	5.17	0.06	74	0	0
ukul.za	*NA*	*NA*	*NA*	*NA*	5.68	0.15	56	0	0
unc.us	474.8	62.7	1.78	0.62	5.35	0.19	280	0	0
valm.ch	1433.0	442.9	6.53	2.40	5.50	0.27	188	0	0

Table S9: Adjusted P-values of linear models for correlations between biotic and abiotic variables and the abundance of genera. Taxa were selected based on their visualization in the constrained ordination Figure S2. P-values are corrected for multiple testing of environmental variables per genus, according to Benjamini-Hochberg.

Genus	soil_pH	ppm_Ca	ppm_Cu	Precip_COLD_Q	Annual_grass
Azorhizobium	0.068	0.068	0.068	**<0.001**	0.570
Bradyrhizobium	**0.020**	**0.004**	**0.004**	0.700	0.563
Calothrix	**<0.001**	**<0.001**	0.053	0.188	0.230
Geobacter	0.525	0.888	**0.013**	0.900	0.567
Skermanella	0.060	0.052	**<0.001**	0.320	**<0.001**
unclass_Burkholderiales	0.088	0.175	0.088	**<0.001**	0.350

mean precipitation in the coldest quarter (Precip_COLD_Q)
annual grass cover (Annual_grass)

Supplementary References

Angel, R., Nepel, M., Panhölzl, C., Schmidt, H., Herbold, C. W., Eichorst, S. A., et al. (2018). Evaluation of primers targeting the diazotroph functional gene and development of NifMAP – a bioinformatics pipeline for analyzing *nifH* amplicon data. *Front. Microbiol.* 9, 703. doi:10.3389/fmicb.2018.00703.
